# Transcriptomic Analysis of Hidradenitis Suppurativa: A Unique Molecular Signature with Broad Immune Activation

**DOI:** 10.3390/ijms242317014

**Published:** 2023-11-30

**Authors:** Hakim Ben Abdallah, Anne Bregnhøj, Lars Iversen, Claus Johansen

**Affiliations:** Department of Dermatology and Venereology, Aarhus University Hospital, 8200 Aarhus, Denmark; annebreg@rm.dk (A.B.); lars.iversen@clin.au.dk (L.I.); claus.johansen@clin.au.dk (C.J.)

**Keywords:** hidradenitis suppurativa, acne inversa, psoriasis, atopic dermatitis, RNA sequencing

## Abstract

Hidradenitis suppurativa is a chronic inflammatory skin disease with limited treatment options. The poorly understood pathogenesis hinders the development of effective treatments; therefore, a pressing need exists to further elucidate the molecular mechanisms in hidradenitis suppurativa. This study investigated the underlying inflammatory pathways and cell types in hidradenitis suppurativa using transcriptomic approaches with RNA sequencing of lesional and non-lesional skin biopsies from hidradenitis suppurativa, which was jointly analyzed with previously published transcriptomic data from atopic dermatitis and psoriasis patients. The differential expression and pathway enrichment analyses demonstrated the activation of multiple inflammatory processes, including the innate and adaptive immune systems, implicated in the hidradenitis suppurativa pathogenesis. In agreement, hidradenitis suppurativa exhibited a unique and heterogeneous cell type signature involving lymphoid and myeloid cells such as B cells and macrophages. Furthermore, hidradenitis suppurativa displayed increased expression of T_H_1/2/17 signatures with no predominant T_H_ signatures unlike psoriasis (T_H_1/17) and atopic dermatitis (T_H_2). In summary, our study provides molecular insights into the pathomechanisms in hidradenitis suppurativa, revealing a strong and widespread immune activation, which may benefit from treatment strategies offering a broad immunomodulation of various key inflammatory pathways. Our data not only corroborate previously reported findings but also enhance our understanding of the immune dysregulation in hidradenitis suppurativa, uncovering novel and potential therapeutic targets.

## 1. Introduction

Hidradenitis suppurativa (HS) is a complex, chronic inflammatory disease with an estimated prevalence of approximately 1% [1]. The disease usually presents in intertriginous body sites (e.g., axillae and groins) with heterogeneous lesions including painful inflammatory nodules and abscesses that may progress to fistulas, sinus tracts, and disfiguring scarring [2]. Despite the relatively high prevalence and severe impact on quality of life, the pathophysiology of HS remains poorly understood, hindering advances in therapeutics [3]. It has been proposed that follicular occlusion, progressing to follicle rupture releasing hair keratins and microbes into the dermis, elicits an aberrant immune response leading to the lesions implicated in HS [4,5]. In contrast to other inflammatory skin disorders such as psoriasis, recent drug development has not enabled highly efficacious therapies for HS [6]. Although biologics appear to be the most effective medical treatment, the efficacy is limited, with few patients achieving almost clear skin [7,8]. To date, adalimumab (TNF inhibitor) and secukinumab (IL-17A inhibitor) have been approved for moderate-to-severe HS [9,10].

In recent years, several studies have explored the alterations of the transcriptome in HS [11,12,13,14,15,16,17]. These studies have provided transcriptome-based insights into multiple genes and pathways that are dysregulated in HS including antimicrobial peptides (e.g., *DEFB4A*) [13,15,16], cytokines (e.g., *IL17A*, *IL17F*, *IL36A*, and *IL36G*) [12,16], T cell and B cell activation [16], and involvement of apocrine sweat glands [13,15].

Given the limited treatment options and the relatively small number of transcriptome studies, further research is urgently needed to elucidate the molecular mechanisms in HS. This study aimed to enhance our understanding of the immunopathogenesis, which may guide the expanding drug development. In this paper, we performed a transcriptomic analysis using RNA sequencing data of HS, atopic dermatitis, and psoriasis patients that allowed us to identify the underlying inflammatory pathways and cell types in HS, providing a molecular rationale for immunomodulatory treatment strategies in HS.

## 2. Results

### 2.1. HS Is Characterized by a Complex and Heterogeneous Activation of the Immune System

**Figure 1:**
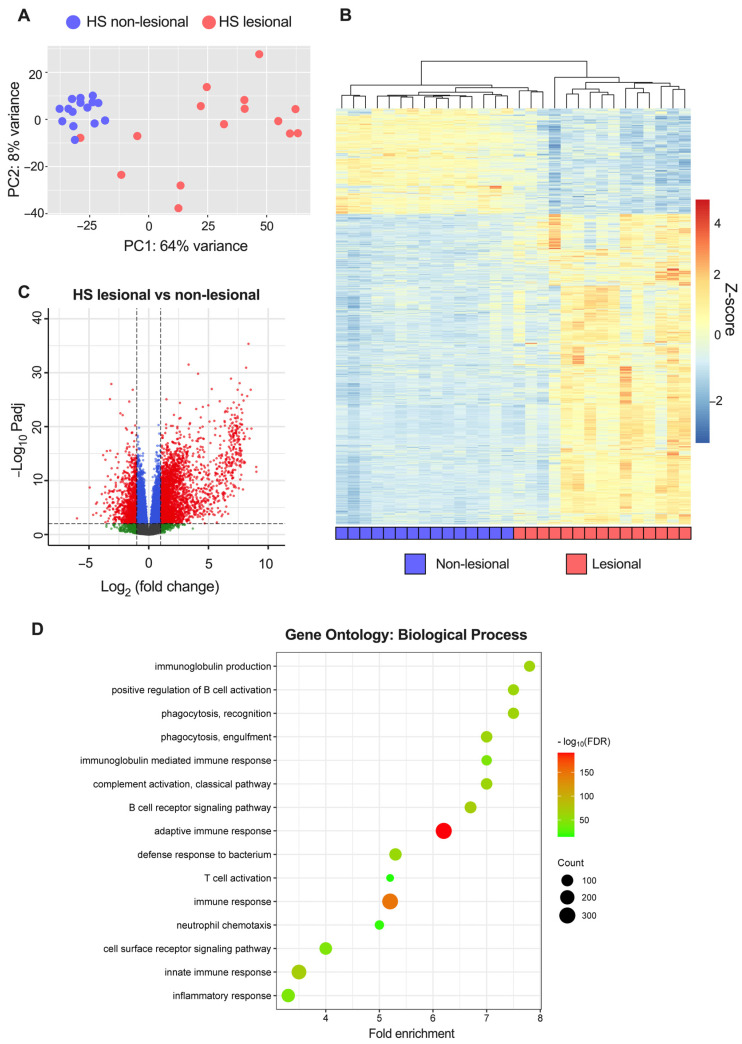
RNA sequencing analysis of hidradenitis suppurativa (HS) lesional and non-lesional skin from 15 patients: (**A**) Principal component analysis of variance stabilizing transformed (vst) data. (**B**) Heatmap of differentially expressed genes (DEGs; |log_2_[fold change]| > 1 and FDR-adjusted *p*-value < 0.01) comparing lesional with non-lesional skin. The genes were filtered to include those with a mean of normalized counts > 10. (**C**) Volcano plot of differential gene expression. The dashed horizontal lines depict an FDR-adjusted *p*-value of 0.01 and the vertical lines depict a log_2_(fold change) of 1. Red dots represent DEGs; blue dots, |log_2_(fold change)| < 1 and P_adj_ < 0.01; green dots, |log_2_(fold change)| > 1 and P_adj_ > 0.01; grey dots, |log_2_(fold change)| < 1 and P_adj_ > 0.01. (**D**) Enrichment analysis of up-regulated DEGs in HS lesional skin showing the top 15 significant GO biological processes terms. Abbreviations: DEGs, differentially expressed genes. HS, hidradenitis suppurativa.

**Figure 2:**
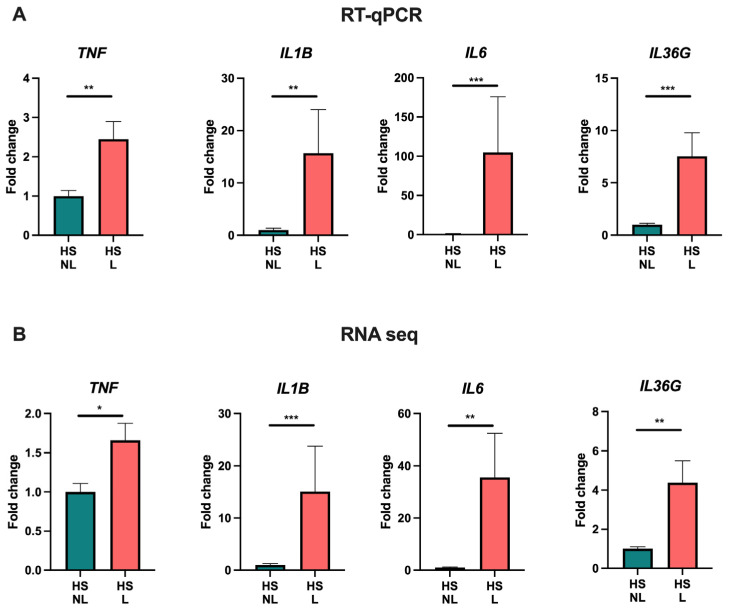
RT-qPCR (**A**) and RNA sequencing analysis (**B**) of key cytokine gene expression in hidradenitis suppurativa skin (*n* = 15). Data are shown as fold change relative to hidradenitis suppurativa non-lesional skin and presented as mean ± SEM. * *p* < 0.05, ** *p* ≤ 0.01, *** *p* ≤ 0.001. Abbreviations: HS, hidradenitis suppurativa. L, lesional. NL, non-lesional.

To explore the transcriptomic alterations in HS, bulk RNA sequencing was performed. When comparing HS lesional with non-lesional skin, 3654 DEGs were detected of which 2636 were upregulated and 1018 were downregulated (Table S1). The most upregulated genes were immunoglobins (e.g., *IGLV4-60* and *IGLV7-46*), matrix metallopeptidases (*MMP3/9*), antimicrobial peptides (*DEFB4A/B* and *S100A7A*), immune-related genes (*IFNγ*, *IL6*, *IL17A/F*, *IL21*, *IL24*, *IL36A*, *CD19/79A*, and *CXCL1/6/13*), and skin-structure-related genes (*KRT13/16*, *SPRR2*, and *SERPINB4*). A principal component analysis and a hierarchically clustered heat map of the DEGs demonstrated an almost complete separation of HS lesional and non-lesional skin, but a heterogenous expression pattern was detected among the HS lesional skin samples (Figure 1A,B). Moreover, volcano plots (HS lesional versus non-lesional skin) visually demonstrated a predominant shift of DEGs to the right, indicating a greater upregulation of DEGs in both the number of genes and the magnitude of gene expression change than down-regulated DEGs (Figure 1C). To examine the functions of the upregulated DEGs, a Gene Ontology (GO) enrichment analysis was performed (Figure 1D). The top 15 significant GO biological process terms were all related to inflammation, highlighting the importance of inflammatory processes implicated in the pathogenesis of HS. Of note, multiple different inflammatory processes were involved in the disease pathogenesis including adaptive immune response (adjusted *p* = 1.3 × 10^−191^), innate immune response (adjusted *p* = 2.4 × 10^−69^), complement activation (adjusted *p* = 4.7 × 10^−63^), positive regulation of B cell activation (adjusted *p* = 1.2 × 10^−61^), defense response to bacterium (adjusted *p* = 1.5 × 10^−59^), neutrophil chemotaxis (adjusted *p* = 8.1 × 10^−20^), and T cell activation (adjusted *p* = 1.2 × 10^−15^). RT-qPCR validation of key immune biomarkers (*TNF*, *IL1B*, *IL6*, and *IL36G*) was performed and showed strong consistency with the results from RNA sequencing (Figure 2). A list of all genes from the differential expression analysis is available in Table S2.

### 2.2. HS Exhibits a Unique Cell Type Signature including Lymphoid Cells (T Cells and B Cells) and Myeloid Cells (Macrophages and Neutrophils)

**Figure 3:**
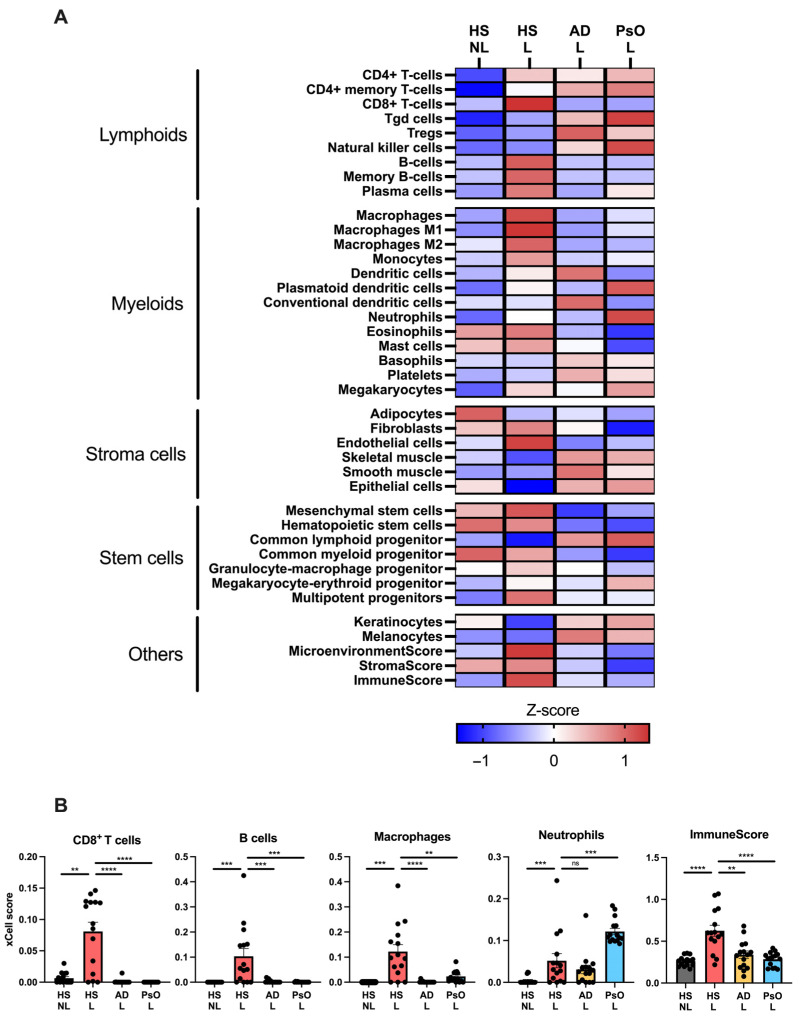
Cell type enrichment analysis using xCell and RNA sequencing data from hidradenitis suppurativa lesional (HS L), HS non-lesional (HS NL), psoriasis lesional (PsO L), and atopic dermatitis lesional (AD L) skin biopsies: (**A**) Heatmap of xCell enrichment z-scores for relevant cell types. (**B**) Graphs with xCell enrichment scores of CD8^+^ T cells, B cells, macrophages, neutrophils, and ImmuneScore (a composite score of immune cells). Data are shown as mean ± SEM. ** *p* ≤ 0.01, *** *p* ≤ 0.001, **** *p* ≤ 0.0001. ns, not significant. Abbreviations: AD, atopic dermatitis. HS, hidradenitis suppurativa. L, lesional. NL, non-lesional. PsO, psoriasis.

**Figure 4:**
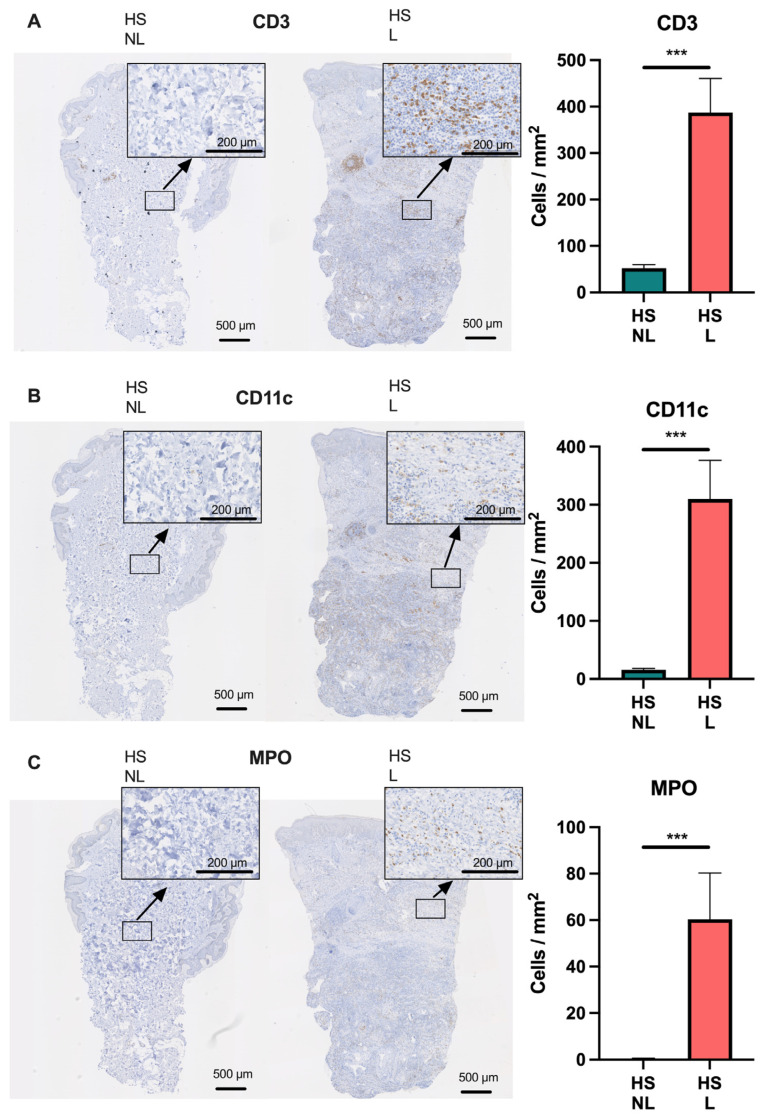
Immunohistochemical analysis of (**A**) CD3^+^, (**B**) CD11c^+^, and (**C**) myeloperoxidase^+^ cells in hidradenitis suppurativa skin (*n* = 15). Data are shown as mean ± SEM. *** *p* ≤ 0.001. Abbreviations: HS, hidradenitis suppurativa. L, lesional. MPO, myeloperoxidase. NL, non-lesional.

To further characterize the transcriptome of HS, a comparison with psoriasis and atopic dermatitis using RNA seq data was performed because the molecular mechanisms in these two diseases have been extensively investigated leading to several approved targeted therapies also undergoing evaluation for HS [18]. To evaluate the major cell types involved in HS, cell-type-signature enrichment scores were estimated and visualized (Figure 3A). Many cell-type signatures were enriched in HS lesional skin compared with non-lesional skin including CD8^+^ T cells (*p* = 0.0012), B cells (*p* = 0.0010), macrophages (*p* = 0.0005), and neutrophils (*p* = 0.0005; Figure 3A,B). Notably, the cell signatures for CD8^+^ T cells, B cells, and macrophages were also significantly enriched in HS compared with psoriasis or atopic dermatitis, whereas the neutrophil signature was less enriched in HS compared with psoriasis (*p* = 0.0008). Moreover, the ImmuneScore (an enrichment score for immune cells) was significantly increased in HS lesional skin compared with HS non-lesional skin (*p* < 0.0001), atopic dermatitis (*p* = 0.0021), and psoriatic skin (*p* < 0.0001), suggesting an activation of a broad range of immune cells in HS. These data demonstrate that HS lesional skin exhibits a unique and more heterogeneous immune cell type signature compared with psoriasis and atopic dermatitis. Consistent with the findings of xCell, immunohistochemical analysis confirmed a significantly increased number of T cells (CD3^+^ cells), macrophages/dendritic cells (CD11c^+^ cells), and neutrophils (myeloperoxidase^+^ cells) in HS lesional skin compared with non-lesional skin (Figure 4).

### 2.3. HS Displays an Inflammatory Pathway Signature Distinct from Psoriasis and Atopic Dermatitis

**Figure 5:**
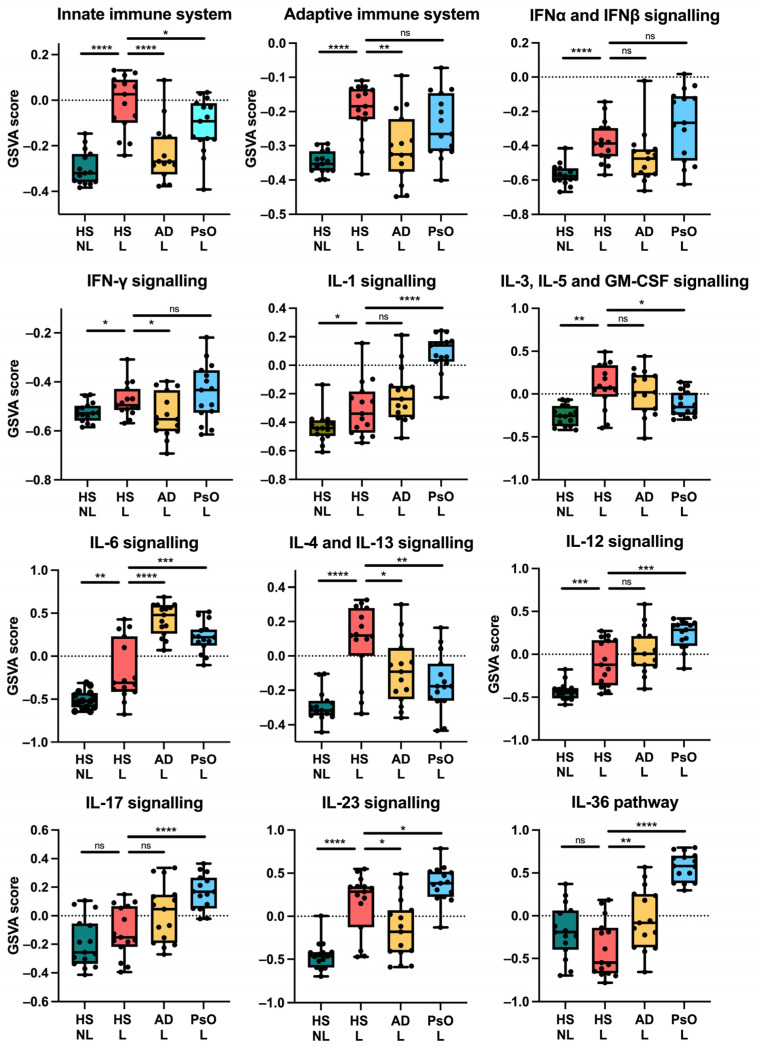
Gene set variation analysis of key immune pathways using RNA sequencing data and Reactome gene sets. * *p* < 0.05, ** *p* ≤ 0.01, *** *p* ≤ 0.001, **** *p* ≤ 0.0001. ns, not significant. Abbreviations: AD, atopic dermatitis. HS, hidradenitis suppurativa. L, lesional. NL, non-lesional. PsO, psoriasis.

**Figure 6:**
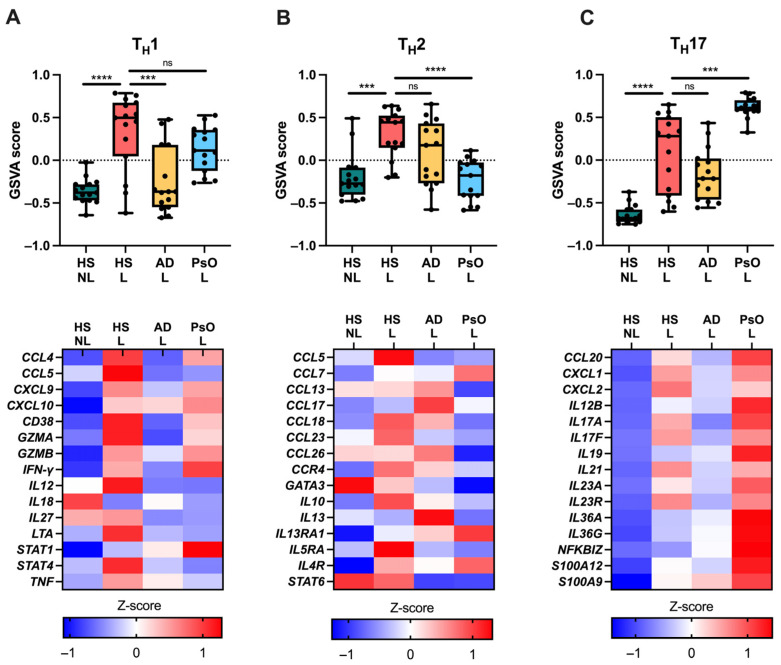
Immune gene signatures using RNA sequencing data. Gene set variation analysis scores (upper panels) and gene expression (lower panels) of (**A**) T_H_1-, (**B**) T_H_2-, and (**C**) T_H_17-related genes. *** *p* ≤ 0.001, **** *p* ≤ 0.0001. ns, not significant. Abbreviations: AD, atopic dermatitis. GSVA, gene set variation analysis. HS, hidradenitis suppurativa. L, lesional. NL, non-lesional. PsO, psoriasis.

To identify the inflammatory pathways underlying HS, we performed a gene set variation analysis (GSVA) for major immune pathways using a subset of relevant Reactome gene sets. The adaptive immune system was significantly enriched in HS lesional skin compared with HS non-lesional skin (*p* < 0.0001) and AD (*p* = 0.0027) but not compared with psoriasis (*p* = 0.1679). Interestingly, the innate immune system enrichment was significantly highest in HS lesional skin (Figure 5). Furthermore, multiple immune pathways were significantly upregulated in HS lesional skin compared with non-lesional skin including IFNα/β (*p* < 0.0001), IFNγ (*p* = 0.0128), IL-1 (*p* = 0.0310), IL-3/IL-5/GM-CSF (*p* = 0.0010), IL-6 (*p* = 0.0038), IL-4/IL-13 (*p* < 0.0001), IL-12 (*p* = 0.0009), and IL-23 (*p* < 0.0001). While the IL-17 (*p* = 0.2429) and IL-36 pathways (*p* = 0.0861) were not significantly altered, the gene expression of *IL17A/F* and *IL36A/G* (FDR < 0.0001) were significantly increased in HS lesional skin compared with non-lesional skin (Figure 6, Table S1). The enrichment of the immune pathways was also high in atopic dermatitis or psoriasis, but the IL-3/IL-5/GM-CSF and IL-4/IL-13 signaling was highest in HS lesional skin.

Moreover, GSVA analysis of T_H_ signatures showed significant upregulation of T_H_1, T_H_2, and T_H_17 in HS lesional skin versus non-lesional skin (*p* < 0.001; Figure 6). The T_H_1 signature was highest in HS lesional and psoriatic skin; T_H_2 was highest in HS lesional skin and atopic dermatitis; and T_H_17 was highest in psoriasis.

Taken together, HS exhibited increased expression of T_H_1/2/17 signatures with no dominance toward a T_H_ signature unlike psoriasis (T_H_1/17) and atopic dermatitis (T_H_2). Moreover, the innate immune system and the IL-3/IL-5/GM-CSF pathway involving myeloid cells such as macrophages and granulocytes were preferentially upregulated in HS, suggesting a widespread activation of the immune system.

## 3. Discussion

Our results identified a unique molecular signature in HS reflecting extensive immune activation of both the adaptive and innate immune systems. In contrast, atopic dermatitis and psoriatic skin exhibited more narrowly dominant immune pathways and cell types. These findings suggest that therapeutics with broad immunomodulation targeting multiple key pathways may increase the chances of managing HS. Other approaches may include stratifying the heterogeneous clinical and molecular presentation of HS to more homogenous subtypes, which hopefully will enable better treatment responses to medical interventions; or targeted therapy addressing the causative and initiating processes of the pathogenesis, although these for now remain unknown.

Despite the heterogeneous molecular profile of HS, our findings are in good agreement with two recent meta-analyses of transcriptomic HS datasets, in which highly significantly upregulated DEGs included immunoglobins, matrix metallopeptidase family genes (e.g., *MMP1/3/9*), antimicrobial peptides (e.g., *DEFB4A/B*), and immune-related genes (e.g., *IFNγ*, *IL1B*, *IL6*, *IL17A/F*, *IL36A/G*, and *CD19*) [13,14].

Consistent with another study [16], our results demonstrate a lack of a dominant T_H_ cytokine axis in HS, whereas atopic dermatitis (T_H_2) and psoriatic skin (T_H_1/17) exhibited dominant axes, which may explain the relative success of targeting these pathways in atopic dermatitis and psoriasis [19,20]. The benefits of IL-17 inhibition have shown to be relatively modest in HS, whereas IL-23 or IL-36 inhibition failed to demonstrate a significant response in phase 2 trials [8,21,22,23].

Consistent with our analyses, previous studies have observed B cells and plasma cells to be involved in HS [16,17,24,25]. In our analyses, B cell signatures and related genes (e.g., *CD19* and *CD79A*) were significantly increased in HS lesional skin, further confirming alterations of B cells in HS pathogenesis. Notably, a case report demonstrated amelioration of HS by B cell depletion with rituximab [26]; however, several drugs (iscalimab, remibrutinib, ianalumab, and fostamatinib) targeting B cells are under development for HS, which will reveal the feasibility of this approach [27,28].

The complement system, a central part of the innate immune system, has been demonstrated to be activated in HS [16,17,29]. Moreover, it has been hypothesized that complement pathways may be activated by microbiomes in HS, providing a possible link between the innate immune system and microbiome [30]. In accordance, we found that upregulated DEGs were related to the complement system (e.g., *C5AR1/2*, and *CR1*) and significantly enriched the complement pathway in HS lesional skin compared to non-lesional skin. However, the inhibition of C5a, a major neutrophil chemoattractant, by IFX-1 failed to demonstrate a significant difference in hidradenitis suppurativa clinical response 50 (HiSCR_50_) compared with placebo [31]. Yet, a post hoc analysis found a dose-dependent significant reduction in draining fistulas (*p* = 0.0359) [32]. Similarly, another C5a inhibitor, avacopan, failed to demonstrate a significant HiSCR_50_ response in the primary analysis of a Phase 2 study, although a subgroup analysis revealed a significantly greater HiSCR_50_ response in Hurley stage III patients compared with placebo (*p* = 0.0349) [33]. These findings suggest that C5a inhibition may have a beneficial effect in severe HS with sinus tract formation. Another approach to modulating the innate immune system involves targeting neutrophils. In agreement with our findings, neutrophils have been demonstrated to be involved in HS pathogenesis [13,16,25,34,35]. Anumigilimab, an antagonist of granulocyte-colony-stimulating factor (G-CSF), blocking neutrophil migration and function, is under development for HS, but no clinical efficacy data have been published yet [35].

In our analyses, we demonstrated a broad activation of the innate immune system including myeloid cells extending beyond neutrophils such as monocytes and macrophages. Unlike C-CSF, which primarily promotes neutrophils, granulocyte-macrophage-colony-stimulating factor (GM-CSF or CSF2) has a broader range of biological functions promoting cell types such as macrophages/monocytes, neutrophils, eosinophils, basophils, and dendritic cells [36]. Furthermore, we demonstrated that the macrophage signature and the IL-3/IL-5/GM-CSF pathway were significantly enriched in HS lesional skin compared with HS non-lesional and psoriatic skin, and the genes for the GM-CSF receptor (*CSF2RA*) and (*CSFR2RB*) were among the upregulated DEGs in HS lesional versus non-lesional skin (Table S1). These findings suggest that GM-CSF might be a novel target in HS that provides broad immunomodulation. While GM-CSF inhibitors are under development, showing no success in psoriasis but varying success in other inflammatory conditions such as rheumatoid arthritis, this mechanism of action has not been evaluated in hidradenitis suppurativa, warranting further investigation [37,38].

Recently, a novel and potential treatment strategy targeting heat shock protein 90 (HSP90), a common chaperone supporting the function of many proinflammatory proteins, has been shown to provide broad-ranging immunomodulation in skin inflammation by targeting multiple inflammatory pathways [39,40,41,42]. Interestingly, a placebo-controlled randomized trial (n = 15) discovered that HSP90 inhibition by RGRN-305 improved both physician- and patient-reported outcomes in HS patients, suggesting that HSP90 may be a novel drug target in HS, though further research is warranted [43].

Our analysis showed that the IL-4 and IL-13 Reactome pathway was significantly enriched in HS lesional skin compared with HS non-lesional skin, atopic dermatitis, and psoriatic skin. Similarly, a meta-analysis of transcriptomic datasets found the IL-4 and IL-13 Reactome pathway to be among the top 20 significant pathways in HS lesional versus non-lesional skin [14]. However, in our analysis, *IL13* was downregulated in HS lesional skin, consistent with other studies [16,44,45], whereas *IL4* was filtered out due to low expression and therefore not captured in our dataset. These findings suggest that genes other than *IL4* and *IL13* were increased in the IL-4 and IL-13 Reactome gene set, reducing the relevancy of this pathway enrichment. Nonetheless, patients with AD have 5.6-fold increased odds for HS, and three cases of HS patients with concomitant AD were successfully treated with dupilumab [45,46,47,48,49]. Thus, the role of IL-4 and IL-13 in HS pathogenesis remains unclear.

While HS is not considered an infectious disease, bacteria may contribute to maintaining inflammation in established lesions [50,51]. Interestingly, defense responses to bacterium (adjusted *p* = 1.5 × 10^−59^) were among the top 15 significantly enriched upregulated GO terms in HS lesional skin, supporting the role of microorganisms in the disease pathogenesis. This is consistent with other transcriptomic studies finding significantly enriched GO terms related to bacteria [13,15]. In accordance, antibiotics can ameliorate HS, suggesting that treatment strategies in HS may benefit from antibacterial effects in addition to immunomodulation [52,53,54].

HS lesional skin is characterized by a heterogenous molecular profile as demonstrated by the high variation in the principal component analysis plot in this study, which is consistent with other studies [16,17,55]. This variation may reflect differences among patients (i.e., inter-individual differences), but affected skin may also vary in the same patient (i.e., intra-individual differences). This heterogeneity contributes to the complexity of HS at the molecular level. Further research involving numerous patients is warranted to investigate whether HS may be stratified into subtypes that exhibit more homogenous clinical and molecular presentations.

Limitations of our study include a lack of biopsies from perilesional lesions and other active lesions besides inflammatory nodules such as tunnels, which likely exhibit different gene expression profiles. Moreover, our patients had moderate-to-severe HS, which may reduce the generalizability to mild HS. Another limitation includes differences in study protocols from biopsy collection to RNA sequencing; however, this has unlikely influenced the results substantially.

In summary, our study provides molecular insights into HS pathogenesis, identifying a strong and heterogeneous immune activation that may benefit from treatment strategies with broad immunomodulation targeting multiple pathways. The data presented here confirm previously reported findings and further characterize the immune dysregulation in HS, revealing novel and potential therapeutic targets.

## 4. Materials and Methods

### 4.1. Biopsies

Three-millimeter punch biopsies were collected from HS lesional skin (edge of an inflammatory nodule) and non-lesional skin (uninvolved skin 10 cm from the active lesion) from 15 patients and stored in liquid nitrogen. The biopsies were obtained from the inguinal or axillary regions. Before the collection of the biopsies, the patients did not receive any systemic or topical treatment for four and two weeks, respectively. The patients were recruited from the outpatient clinic at the Department of Dermatology and Venereology, Aarhus University Hospital in Denmark. Written informed consent was obtained from the patients. The study protocol was approved by the Central Denmark Regional Ethical Committee (no. 1-10-72-48-21).

### 4.2. RNA Isolation

Upon RNA isolation, the biopsies were transferred to RNAlater-ICE (ThermoFisher Scientific, Waltham, MA, USA) at −20 °C overnight. Then, the biopsies were added to SV RNA lysis buffer with β-mercaptoethanol (SV Total RNA Isolation System; Promega, Madison, WI, USA) followed by homogenization using TissueLyser (Qiagen, Hilden, Germany). The remaining steps of RNA isolation were performed per the manufacturer’s instructions. The RNA quantity was measured using Nanodrop 2000 (ThermoFisher), whereas the RNA quality was determined using Bioanalyzer 2100 (Agilent Technologies, Santa Clara, CA, USA).

### 4.3. RNA Sequencing

Paired-end RNA sequencing was performed per the protocols of Eurofins Genomics Europe Sequencing GmbH (Konstanz, Germany) as previously described [39]. In brief, sequencing libraries were generated using NEBNext Ultra II Directional RNA Library Prep Kit and sequenced using Illumina NovaSeq 6000 platform for at least 20 million read pairs. Raw RNA seq data from psoriasis (*n* = 15) and atopic dermatitis (*n* = 15) patients were obtained from a previously published study available at Gene Expression Omnibus (GSE 121212) [18,56]. Quality control of raw sequence data was performed using FastQC (version 0.12.0). Reads were aligned to the human reference genome (GRCh38.p14 primary assembly) using the R package Subread (version 2.10.3) [57]. Uniquely aligned unambiguous reads were used for the subsequent analyses. Lowly expressed genes with less than five samples with normalized counts greater or equal to five were filtered out. Variance stabilizing transformed (VST) data using DESeq2 (1.36.0) were used for downstream analyses such as principal component analysis and heat map (pheatmap 1.0.12) [58]. Differential expression analyses were performed with DESeq2 using the Wald test [58]. False discovery rate (FDR)-adjusted *p*-values ≤ 0.01 and |log2(fold change)| ≥ 1 were significance thresholds for differentially expressed genes (DEGs). Gene Ontology (GO) enrichment analyses were performed with DAVID and validated with ShinyGO 0.76 [59,60]. Cell types enrichment analyses were performed on TPM (transcripts per million kilobase) normalized dataset using xCell webtool, a bioinformatics tool that generates enrichment scores for 64 cell types based on gene expression data [61]. Gene set variation analyses were performed with the R package GSVA (version 1.46.0) using VST normalized dataset [62]. Reactome gene sets were imported from The Molecular Signatures Database (MSigDB) through msigbr (version 7.5.1), whereas gene sets for T_H_ type (T_H_1, T_H_2, T_H_17) were based on previously published studies [63,64,65].

### 4.4. RT-qPCR

Reverse transcription of total RNA to cDNA was performed with TaqMan Reverse Transcription Reagents (ThermoFisher) and Peltier Thermal Cycler-200 (MJ Research Inc., Waltham, MA, USA) per the manufacturer’s instructions. Real-time PCR was performed with 10 ng of cDNA (20 µL reactions) in triplicates using StepOnePlus Real-Time PCR system (ThemoFisher) and TaqMan Universal PCR Master Mix and primers/probes (ThermoFisher) for *TNF* (Hs00174128_m1), *IL1B* (Hs01555410_m1), *IL6* (Hs00174131_m1), *IL36G* (Hs00219742_m1), and *RPLP0* (Hs99999902_m1). The real-time PCR consisted of 2 consecutive steps (2 min at 50 °C and 10 min at 95 °C) followed by 40 cycles (15 s at 95 °C and 1 min at 60 °C). Normalized gene expression values were determined with the standard curve method using StepOne Software v2.1 and *RPLP0* as reference gene [66].

### 4.5. Histology

Three-millimeter punch biopsies were fixed in 4% formaldehyde and embedded in paraffin. Then, 4 µm sections were sliced and immunohistochemically stained (IHC) using primary antibodies ((catalog#) CD3 (103R-96), CD11c (EP157), and myeloperoxidase (SP72)) purchased from Cell Marque, Rocklin, CA, USA. Heat-induced antigen retrieval was performed (20 min at 97 °C) in Tris-EGTA buffer (pH 9). A polymer detection system was used for detection (Quanto Detection System, ThermoFisher). The sections were counterstained with hematoxylin. All slides were scanned with 20× objective using the whole slide scanner NanoZoomer 2.0-HT (Hamamatsu Photonics K.K, Hamamatsu City, Japan). Quantitative image analysis was performed in QuPath 0.4.2 using the Cell Detection command with default settings followed by a manual review and corrections [67].

### 4.6. Statistics

For single-group comparisons between HS lesional and non-lesional samples, two-tailed paired Student’s *t*-tests were used for normally distributed data, while Wilcoxon signed-rank tests were used for non-normally distributed data. When comparing single-group data between HS, atopic dermatitis, and psoriasis samples, two-tailed Student’s *t*-tests were applied for normally distributed data, and Mann–Whitney tests were used for non-normally distributed data. *p*-values < 0.05 were considered significant. All statistical analyses were performed with GraphPad Prism 10.0.1 and R 4.2.0 software.

## Data Availability

The data are available at Gene Expression Omnibus (GSE249027 (https://www.ncbi.nlm.nih.gov/geo/query/acc.cgi?acc=GSE249027 (accessed on 25 November 2023))).

## Supplementary Materials

The following supporting information can be downloaded at: https://www.mdpi.com/article/10.3390/ijms242317014/s1.

## References

[1] Nguyen T.V., Damiani G., Orenstein L.A.V., Hamzavi I., Jemec G.B. (2021). Hidradenitis suppurativa: An update on epidemiology, phenotypes, diagnosis, pathogenesis, comorbidities and quality of life. J. Eur. Acad. Dermatol. Venereol..

[2] Jemec G.B. (2012). Clinical practice. Hidradenitis suppurativa. N. Engl. J. Med..

[3] Sabat R., Jemec G.B.E., Matusiak Ł., Kimball A.B., Prens E., Wolk K. (2020). Hidradenitis suppurativa. Nat. Rev. Dis. Primers.

[4] Goldburg S.R., Strober B.E., Payette M.J. (2020). Hidradenitis suppurativa: Epidemiology, clinical presentation, and pathogenesis. J. Am. Acad. Dermatol..

[5] Molinelli E., Gioacchini H., Sapigni C., Diotallevi F., Brisigotti V., Rizzetto G., Offidani A., Simonetti O. (2023). New Insight into the Molecular Pathomechanism and Immunomodulatory Treatments of Hidradenitis Suppurativa. Int. J. Mol. Sci..

[6] Ben Abdallah H., Johansen C., Iversen L. (2021). Key Signaling Pathways in Psoriasis: Recent Insights from Antipsoriatic Therapeutics. Psoriasis.

[7] Kimball A.B., Okun M.M., Williams D.A., Gottlieb A.B., Papp K.A., Zouboulis C.C., Armstrong A.W., Kerdel F., Gold M.H., Forman S.B. (2016). Two Phase 3 Trials of Adalimumab for Hidradenitis Suppurativa. N. Engl. J. Med..

[8] Kimball A.B., Jemec G.B.E., Alavi A., Reguiai Z., Gottlieb A.B., Bechara F.G., Paul C., Giamarellos Bourboulis E.J., Villani A.P., Schwinn A. (2023). Secukinumab in moderate-to-severe hidradenitis suppurativa (SUNSHINE and SUNRISE): Week 16 and week 52 results of two identical, multicentre, randomised, placebo-controlled, double-blind phase 3 trials. Lancet.

[9] AbbVie AbbVie’s HUMIRA^®^ (Adalimumab) Receives First and Only U.S. Food and Drug Administration Approval for Moderate to Severe Hidradenitis Suppurativa. https://news.abbvie.com/news/abbvies-humira-adalimumab-receives-first-and-only-us-food-and-drug-administration-approval-for-moderate-to-severe-hidradenitis-suppurativa.htm.

[10] Novartis Novartis Receives European Approval for Cosentyx® as First and Only IL-17A Inhibitor for Hidradenitis Suppurativa. https://www.novartis.com/news/media-releases/novartis-receives-european-approval-cosentyx-first-and-only-il-17a-inhibitor-hidradenitis-suppurativa.

[11] Shanmugam V.K., Jones D., McNish S., Bendall M.L., Crandall K.A. (2019). Transcriptome patterns in hidradenitis suppurativa: Support for the role of antimicrobial peptides and interferon pathways in disease pathogenesis. Clin. Exp. Dermatol..

[12] Rumberger B.E., Boarder E.L., Owens S.L., Howell M.D. (2020). Transcriptomic analysis of hidradenitis suppurativa skin suggests roles for multiple inflammatory pathways in disease pathogenesis. Inflamm. Res..

[13] Freudenberg J.M., Liu Z., Singh J., Thomas E., Traini C., Rajpal D.K., Sayed C.J. (2023). A Hidradenitis Suppurativa molecular disease signature derived from patient samples by high-throughput RNA sequencing and re-analysis of previously reported transcriptomic data sets. PLoS ONE.

[14] de Oliveira A., Bloise G., Moltrasio C., Coelho A., Agrelli A., Moura R., Tricarico P.M., Jamain S., Marzano A.V., Crovella S. (2022). Transcriptome Meta-Analysis Confirms the Hidradenitis Suppurativa Pathogenic Triad: Upregulated Inflammation, Altered Epithelial Organization, and Dysregulated Metabolic Signaling. Biomolecules.

[15] Coates M., Mariottoni P., Corcoran D.L., Kirshner H.F., Jaleel T., Brown D.A., Brooks S.R., Murray J., Morasso M.I., MacLeod A.S. (2019). The skin transcriptome in hidradenitis suppurativa uncovers an antimicrobial and sweat gland gene signature which has distinct overlap with wounded skin. PLoS ONE.

[16] Gudjonsson J.E., Tsoi L.C., Ma F., Billi A.C., van Straalen K.R., Vossen A., van der Zee H.H., Harms P.W., Wasikowski R., Yee C.M. (2020). Contribution of plasma cells and B cells to hidradenitis suppurativa pathogenesis. JCI Insight.

[17] Lowe M.M., Naik H.B., Clancy S., Pauli M., Smith K.M., Bi Y., Dunstan R., Gudjonsson J.E., Paul M., Harris H. (2020). Immunopathogenesis of hidradenitis suppurativa and response to anti-TNF-α therapy. JCI Insight.

[18] Tsoi L.C., Rodriguez E., Degenhardt F., Baurecht H., Wehkamp U., Volks N., Szymczak S., Swindell W.R., Sarkar M.K., Raja K. (2019). Atopic Dermatitis Is an IL-13-Dominant Disease with Greater Molecular Heterogeneity Compared to Psoriasis. J. Investig. Dermatol..

[19] Sbidian E., Chaimani A., Garcia-Doval I., Doney L., Dressler C., Hua C., Hughes C., Naldi L., Afach S., Le Cleach L. (2022). Systemic pharmacological treatments for chronic plaque psoriasis: A network meta-analysis. Cochrane Database Syst. Rev..

[20] Silverberg J.I., Thyssen J.P., Fahrbach K., Mickle K., Cappelleri J.C., Romero W., Cameron M.C., Myers D.E., Clibborn C., DiBonaventura M. (2021). Comparative efficacy and safety of systemic therapies used in moderate-to-severe atopic dermatitis: A systematic literature review and network meta-analysis. J. Eur. Acad. Dermatol. Venereol..

[21] Kimball A.B., Prens E.P., Passeron T., Maverakis E., Turchin I., Beeck S., Drogaris L., Geng Z., Zhan T., Messina I. (2023). Efficacy and Safety of Risankizumab for the Treatment of Hidradenitis Suppurativa: A Phase 2, Randomized, Placebo-Controlled Trial. Dermatol. Ther..

[22] Janssen Research & Development, L. A Study to Evaluate the Efficacy, Safety, and Tolerability of Guselkumab for the Treatment of Participants With Moderate to Severe Hidradenitis Suppurativa (HS) (NOVA). https://classic.clinicaltrials.gov/ct2/show/NCT03628924.

[23] Anaptys Bio I. Anaptysbio Reports Harp Phase 2 Top-Line Data of Imsidolimab in Moderate-to-Severe Hidradenitis Suppurativa. https://ir.anaptysbio.com/news-releases/news-release-details/anaptysbio-reports-harp-phase-2-top-line-data-imsidolimab.

[24] Musilova J., Moran B., Sweeney C.M., Malara A., Zaborowski A., Hughes R., Winter D.C., Fletcher J.M., Kirby B. (2020). Enrichment of Plasma Cells in the Peripheral Blood and Skin of Patients with Hidradenitis Suppurativa. J. Investig. Dermatol..

[25] Byrd A.S., Carmona-Rivera C., O’Neil L.J., Carlucci P.M., Cisar C., Rosenberg A.Z., Kerns M.L., Caffrey J.A., Milner S.M., Sacks J.M. (2019). Neutrophil extracellular traps, B cells, and type I interferons contribute to immune dysregulation in hidradenitis suppurativa. Sci. Transl. Med..

[26] Takahashi K., Yanagi T., Kitamura S., Hata H., Imafuku K., Iwami D., Hotta K., Morita K., Shinohara N., Shimizu H. (2018). Successful treatment of hidradenitis suppurativa with rituximab for a patient with idiopathic carpotarsal osteolysis and chronic active antibody-mediated rejection. J. Dermatol..

[27] Pharmaceuticals N. Study of Efficacy and Safety of Investigational Treatments in Patients With Moderate to Severe Hidradenitis Suppurativa. https://classic.clinicaltrials.gov/ct2/show/NCT03827798.

[28] Practice H.H.M. Study of the Effect of Fostamatinib upon Cutaneous Inflammation in the Setting of Hidradenitis Suppurativa. https://classic.clinicaltrials.gov/ct2/show/NCT05040698.

[29] Kanni T., Zenker O., Habel M., Riedemann N., Giamarellos-Bourboulis E.J. (2018). Complement activation in hidradenitis suppurativa: A new pathway of pathogenesis?. Br. J. Dermatol..

[30] Grand D., Navrazhina K., Frew J.W. (2020). Integrating complement into the molecular pathogenesis of Hidradenitis Suppurativa. Exp. Dermatol..

[31] InflaRx InflaRx Announces Top-Line SHINE Phase IIb Results for IFX-1 in Hidradenitis Suppurativa. https://www.inflarx.de/Home/Investors/Press-Releases/06-2019-InflaRx-Announces--Top-Line-SHINE-Phase-IIb-Results-for-IFX-1-in-Hidradenitis-Suppurativa-.html.

[32] InflaRx 07-2019-InflaRx Reports Additional Analysis of the SHINE Phase IIb Results for IFX-1 in Hidradenitis Suppurativa. https://www.inflarx.de/Home/Investors/Press-Releases/07-2019-InflaRx-Reports-Additional-Analysis-of-the-SHINE-Phase-IIb-Results-for-IFX-1-in-Hidradenitis-Suppurativa-.html.

[33] Kirby J.S., Prens E., Jemec G.B., Prasad S., Schall T., Staehr P. (2021). LB791 Avacopan, a highly selective small molecule inhibitor of c5a receptor, in patients with Hidradenitis Suppurativa: Initial results from a randomized, double-blind, placebo-controlled, phase 2 study (aurora). J. Investig. Dermatol..

[34] Lima A.L., Karl I., Giner T., Poppe H., Schmidt M., Presser D., Goebeler M., Bauer B. (2016). Keratinocytes and neutrophils are important sources of proinflammatory molecules in hidradenitis suppurativa. Br. J. Dermatol..

[35] Gamell C., Bankovacki A., Scalzo-Inguanti K., Sedgmen B., Alhamdoosh M., Gail E., Turkovic L., Millar C., Johnson L., Wahlsten M. (2023). CSL324, a granulocyte colony-stimulating factor receptor antagonist, blocks neutrophil migration markers that are upregulated in hidradenitis suppurativa. Br. J. Dermatol..

[36] Lazarus H.M., Gale R.P. (2021). G-CSF and GM-CSF Are Different. Which One Is Better for COVID-19?. Acta Haematol..

[37] Papp K.A., Gooderham M., Jenkins R., Vender R., Szepietowski J.C., Wagner T., Hunt B., Souberbielle B. (2019). Granulocyte-macrophage colony-stimulating factor (GM-CSF) as a therapeutic target in psoriasis: Randomized, controlled investigation using namilumab, a specific human anti-GM-CSF monoclonal antibody. Br. J. Dermatol..

[38] Lee K.M.C., Achuthan A.A., Hamilton J.A. (2020). GM-CSF: A Promising Target in Inflammation and Autoimmunity. Immunotargets Ther.

[39] Ben Abdallah H., Seeler S., Bregnhøj A., Ghatnekar G., Kristensen L.S., Iversen L., Johansen C. (2023). Heat shock protein 90 inhibitor RGRN-305 potently attenuates skin inflammation. Front. Immunol..

[40] Bregnhøj A., Thuesen K.K.H., Emmanuel T., Litman T., Grek C.L., Ghatnekar G.S., Johansen C., Iversen L. (2022). HSP90 inhibitor RGRN-305 for oral treatment of plaque-type psoriasis: Efficacy, safety and biomarker results in an open-label proof-of-concept study. Br. J. Dermatol..

[41] Hansen R.S., Thuesen K.K.H., Bregnhøj A., Moldovan L.I., Kristensen L.S., Grek C.L., Ghatnekar G.S., Iversen L., Johansen C. (2021). The HSP90 inhibitor RGRN-305 exhibits strong immunomodulatory effects in human keratinocytes. Exp. Dermatol..

[42] Stenderup K., Rosada C., Gavillet B., Vuagniaux G., Dam T.N. (2014). Debio 0932, a new oral Hsp90 inhibitor, alleviates psoriasis in a xenograft transplantation model. Acta Derm. Venereol..

[43] Ben Abdallah H.B.A., Emmanuel T., Ghatnekar G., Johansen C., Iversen L. (2023). A double-blind, placebo-controlled, randomized trial evaluating the efficacy and safety of a HSP90 inhibitor (RGRN-305) in hidradenitis suppurativa. EADV.org Abstract Book 2023.

[44] Vossen A., van der Zee H.H., Tsoi L.C., Xing X., Devalaraja M., Gudjonsson J.E., Prens E.P. (2019). Novel cytokine and chemokine markers of hidradenitis suppurativa reflect chronic inflammation and itch. Allergy.

[45] Thomi R., Cazzaniga S., Seyed Jafari S.M., Schlapbach C., Hunger R.E. (2018). Association of Hidradenitis Suppurativa With T Helper 1/T Helper 17 Phenotypes: A Semantic Map Analysis. JAMA Dermatol..

[46] Kaakati R.N., Tanaka J., Liu B., Ward R., Macleod A.S., Green C.L., Jaleel T. (2021). Atopic dermatitis is associated with hidradenitis suppurativa diagnosis: A single institution retrospective cohort study. JAAD Int..

[47] Gambardella A., Calabrese G., Di Brizzi E.V., Alfano R., Argenziano G. (2020). A case of Atopic dermatitis and Hidradenitis Suppurativa successfully treated with Dupilumab. J. Eur. Acad. Dermatol. Venereol..

[48] Cho M.K., Shin J.U., Kim D.H., Lee H.J. (2022). Severe atopic dermatitis and concurrent severe hidradenitis suppurativa successfully treated with dupilumab. Clin. Exp. Dermatol..

[49] Molinelli E., Sapigni C., Simonetti O., Radi G., Gambini D., Maurizi A., Rizzetto G., D’Agostino G.M., Offidani A. (2022). Successfully and safety use of dupilumab in the management of severe atopic dermatitis and concomitant moderate-to-severe hidradenitis suppurativa. Dermatol. Ther..

[50] Benzecry V., Grancini A., Guanziroli E., Nazzaro G., Barbareschi M., Marzano A.V., Muratori S., Veraldi S. (2020). Hidradenitis suppurativa/acne inversa: A prospective bacteriological study and review of the literature. G. Ital. Di Dermatol. E Venereol. Organo Uff. Soc. Ital. Di Dermatol. E Sifilogr..

[51] Gierek M., Ochała-Gierek G., Kitala D., Łabuś W., Bergler-Czop B. (2022). Hidradenitis suppurativa: Bacteriological study in surgical treatment. Postep. Dermatol. I Alergol..

[52] Gener G., Canoui-Poitrine F., Revuz J.E., Faye O., Poli F., Gabison G., Pouget F., Viallette C., Wolkenstein P., Bastuji-Garin S. (2009). Combination therapy with clindamycin and rifampicin for hidradenitis suppurativa: A series of 116 consecutive patients. Dermatology.

[53] Join-Lambert O., Coignard H., Jais J.P., Guet-Revillet H., Poirée S., Fraitag S., Jullien V., Ribadeau-Dumas F., Thèze J., Le Guern A.S. (2011). Efficacy of rifampin-moxifloxacin-metronidazole combination therapy in hidradenitis suppurativa. Dermatology.

[54] Molinelli E., De Simoni E., Candelora M., Sapigni C., Brisigotti V., Rizzetto G., Offidani A., Simonetti O. (2023). Systemic Antibiotic Therapy in Hidradenitis Suppurativa: A Review on Treatment Landscape and Current Issues. Antibiotics.

[55] Dudink K., Bouwman K., Chen Y., DePrimo S.E., Munoz-Elias E.J., Aarts P., Schappin R., Florencia E.F., van Heeswijk B., Prens L.M. (2023). Guselkumab for hidradenitis suppurativa: A phase II, open-label, mode-of-action study. Br. J. Dermatol..

[56] Barrett T., Wilhite S.E., Ledoux P., Evangelista C., Kim I.F., Tomashevsky M., Marshall K.A., Phillippy K.H., Sherman P.M., Holko M. (2013). NCBI GEO: Archive for functional genomics data sets--update. Nucleic Acids Res..

[57] Liao Y., Smyth G.K., Shi W. (2019). The R package Rsubread is easier, faster, cheaper and better for alignment and quantification of RNA sequencing reads. Nucleic Acids Res..

[58] Love M.I., Huber W., Anders S. (2014). Moderated estimation of fold change and dispersion for RNA-seq data with DESeq2. Genome Biol..

[59] Ge S.X., Jung D., Yao R. (2020). ShinyGO: A graphical gene-set enrichment tool for animals and plants. Bioinformatics.

[60] Sherman B.T., Hao M., Qiu J., Jiao X., Baseler M.W., Lane H.C., Imamichi T., Chang W. (2022). DAVID: A web server for functional enrichment analysis and functional annotation of gene lists (2021 update). Nucleic Acids Res..

[61] Aran D., Hu Z., Butte A.J. (2017). xCell: Digitally portraying the tissue cellular heterogeneity landscape. Genome Biol..

[62] Hänzelmann S., Castelo R., Guinney J. (2013). GSVA: Gene set variation analysis for microarray and RNA-seq data. BMC Bioinform..

[63] Radens C.M., Blake D., Jewell P., Barash Y., Lynch K.W. (2020). Meta-analysis of transcriptomic variation in T-cell populations reveals both variable and consistent signatures of gene expression and splicing. Rna.

[64] He H., Bissonnette R., Wu J., Diaz A., Saint-Cyr Proulx E., Maari C., Jack C., Louis M., Estrada Y., Krueger J.G. (2021). Tape strips detect distinct immune and barrier profiles in atopic dermatitis and psoriasis. J. Allergy Clin. Immunol..

[65] Suárez-Fariñas M., Ungar B., Noda S., Shroff A., Mansouri Y., Fuentes-Duculan J., Czernik A., Zheng X., Estrada Y.D., Xu H. (2015). Alopecia areata profiling shows TH1, TH2, and IL-23 cytokine activation without parallel TH17/TH22 skewing. J. Allergy Clin. Immunol..

[66] Biosystems A. (2010). Applied Biosystems StepOne™ and StepOnePlus™ Real-Time PCR Systems.

[67] Bankhead P., Loughrey M.B., Fernández J.A., Dombrowski Y., McArt D.G., Dunne P.D., McQuaid S., Gray R.T., Murray L.J., Coleman H.G. (2017). QuPath: Open source software for digital pathology image analysis. Sci. Rep..

